# A Case of Carcinoma of the Lower Jaw—Operation

**Published:** 1900-01

**Authors:** A. Frank Kerns


ARTICLE III.

### A CASE OF CARCINOMA OF THE LOWER JAW—OPERATION.

REPORTED BY A. FRANK KERNS.

[A committee of ten students from the Kansas City Dental College was invited by Dr. J. D. Griffith to be present at the Sisters' Hospital, Kansas City, Mo., at 10 o'clock, Saturday, October 2nd, 1897, to witness an operation to be performed by himself and Dr. J. D. Patterson for carcinoma on the lower maxilla of P. Kilmer, of Humboldt, Kansas. The committee went according to invitation, and found at the hospital Dr. T. G. Vernon, a dentist of Paola, Kansas, to whom we are indebted for at least a portion of the history of the case].

The patient, P. Kilmer, is a white man fifty-two years of age. He has a family consisting of a wife and two children. For the past four years he has lived on a farm, but the twelve years prior to that were spent here in Kansas City in the employ of the Pabst Brewing Co.

His present trouble began about one year ago, the first indications being a soreness and swelling of the lower jaw in the neighborhood of right bicuspids. About that time he happened to be here in the city, and thinking the swelling was caused by an abscessed tooth, he applied to Dr. Buchanan, a dentist, who found both bicuspids sound, yet quite loose. Dr. Buchanan extracted both bicuspids and Mr. Kilmer thought his trouble at an end. After going home, his jaw improved only for a short time and then began to grow worse. He doctored himself at home for a short time without being benefited. The inferior molars on the right side became loose, and he picked them out with his fingers.

About five weeks ago, while passing through Paola, Kansas, he went into the office of T. G. Vernon for examination. Dr. Vernon picked out a few small pieces of necrosed bone, and treated the opening with peroxide of hydrogen and other anti-home on account of pressing. business, he returned in a few days receiving more ease than he had had for several months, although he had not experienced much pain at any time. Having to go home on account of pressing business, he returned in a few days to Paola for further treatment by Dr. Vernon. This time he remained at Paola for two weeks, during which period he seemed to improve. Having to go home again, he returned to Paola in one week. At this time Dr. Vernon took out quite a large piece of detached necrosed bone, and a further examination revealed a pathological fracture of the lower jaw, there being about one inch of the bone gone. The general appearance of the patient showed that he was very much worse, and Dr. Vernon advised him to come to Kansas City for treatment. In company with Dr. Vernon he came to the city October 17th, and was examined by J. D. Patterson, D. D. S. After a diagnosis of the case, Dr. Patterson decided that it was carcinoma; but, that there be no mistake, he sent a specimen to Dr. Newton McVey for microscopical examination. Dr. McVey only corroborated the decision of Dr. Patterson. A surgical operation was advised, and Dr. J. D. Griffith, who stands in the foremost rank as a surgeon, was chosen to perform the operation, Dr. Patterson to assist.

Upon the arrival of the committee at the hospital we found that the instruments needed for the operation were undergoing thorough sterilization by the method usually employed there, which is the use of a hot solution of boracic acid. These instuments were taken from this hot solution and deposited in another antiseptic solution, placing like instruments together, so that no time would be lost in getting the instruments needed. The doctors were very careful also in thoroughly cleansing their hands and making them as near aseptic as possible.

The patient was brought into the room in an unconscious condition, chloroform having been administered by means of the Esmarch inhaler. He was placed upon the operating table on his back, with a pillow under his shoulders and neck so that his head would drop back, thereby preventing blood from passing back into the trachea. Chloroform was then administered by means of a catheter placed in the nose and the air from the chloroform forced through it. Dr. Patterson extracted the remaining teeth on the right side of the lower jaw, consisting of the central, lateral and cuspid. The blood thus caused was taken from the mouth by means of a swab.

Dr. Griffith then passed a threaded needle through the middle of the tongue, by means of which thread the tongue was held forward, preventing its dropping back into the pharynx. He then made an incision from about the lower edge of the orbiculoris oris muscle in front to the point of the chin. An incision was then made to the right near the lower border of the body of the maxilla almost to the angle, thence upward along the ramus to a point in front of the lower part of the ear. In making this incision, and throughout the whole operation, blood-vessels were properly cared for, the principal one being the facial artery. After the healthy tissue had been dissected from the anterior part of the right side of the maxilla, a chain saw was passed around the bone and it was severed at the symphysis. Then the dissecting proceeded back along the body and ramus of the bone, taking with the bone all the diseased soft parts, until within about an inch of the condyle, where the bone was again severed by means of the chain saw, thus leaving only a small part of the right half of the bone. The edges of the remaining portions of the bone where it was severed were carefully smoothed, that the healing of the wound be not retarded, and all cause of irritation removed. While dissecting along the posterior border of the ramus, great care was taken that the carotid artery be not severed. An incision two or three inches in length was then made from about the angle of the jaw down the neck. A small pocket of pus was found underlying the sheath of the sterno-cleido-mastoid muscle. This was taken out and all the parts cleansed as thoroughly as possible. Iodoform was used freely and the wound stitched. There were only four or five stitches needed to close the incision on the inside of the mouth, and these were taken with ligature. Cat-gut was used in stiching together the external parts. Use was made of two drainage-tubes. The patient's head and neck were then bandaged, and he was removed to his room.—*Kansas City Medical Index.*

---

